# Prevalence and characterization of third-generation cephalosporin, carbapenem and colistin-resistant Enterobacterales isolated from clinical samples in Cambodia

**DOI:** 10.1016/j.nmni.2025.101649

**Published:** 2025-10-10

**Authors:** Gauthier Delvallez, Sokleaph Cheng, Florian Girond, Sidonn Krang, Soda Meng, Puthea Nop, Seiha Heng, Samrach Han, Sokunthy Keo, Kunthea Kong, Anne-Laure Bañuls, Mallorie Hide

**Affiliations:** a Medical Biology Laboratory, Institut Pasteur du Cambodge, Phnom Penh, Cambodia; b LMI Drug Resistance in Southeast Asia, Institut Pasteur du Cambodge, Phnom Penh, Cambodia; c Communicable Disease Control Department, Ministry of Health, Phnom Penh, Cambodia; d MIVEGEC, Univ. Montpellier, CNRS, IRD, Montpellier, France

**Keywords:** Antimicrobial resistance (AMR), Cambodia, Enterobacterales, Third generation cephalosporins (3 GC), Extended-spectrum beta-lactamase (ESBL), Carbapenemase producing enterobacterales (CPE), Colistin resistance

## Abstract

**Objectives:**

Despite the threat of antimicrobial resistance (AMR) worldwide, limited studies describe the situation in human infections in Cambodia. Our study aims to evaluate the current state of AMR and describe the resistance mechanisms of the main Enterobacterales responsible for various infections in Cambodia.

**Methods:**

Between January and April 2020, 222 clinical Enterobacterales isolates were collected from routine diagnostics at the Medical Biology Laboratory of the Institut Pasteur du Cambodge, Phnom Penh. Antimicrobial susceptibility testing and resistance phenotype analysis were performed. Beta-lactamase genes *bla*_CTX-M_*, bla*_SHV_*, bla*_TEM_ and *bla*_OXA-1_ were screened in isolates resistant to third generation cephalosporin (3 GC). Carbapenem-resistant isolates were tested for carbapenemase production using a rapid immunochromatographic assay and confirmed by gene detection. Isolates harboring *bla*_CTX-M_ and carbapenemase genes were further characterized by sequencing.

**Results:**

Overall, 39.2 % of isolates were resistant to at least one 3 GC, with 30.2 % confirmed as extended-spectrum beta-lactamase (ESBL) producers. The corresponding ESBL genes pertain to the KLUB/CTX-M-1 and KLUY/CTX-M-9 classes. Carbapenemase genes *bla*_NDM_ and *bla*_OXA–48_ were detected in 4.1 % of isolates. Notably, 6.5 % of ESBL-producing *Escherichia coli* and *Klebsiella pneumoniae* also exhibited colistin resistance.

**Conclusion:**

The prevalence and molecular characterization of 3 GC-resistant and ESBL-producing Enterobacterales in our study is consistent with recent reports. Our findings further confirm the high rate of carbapenemase producing Enterobacterales (NDM and OXA-48), including in community-acquired infections. Additionally, we report notable colistin resistance among ESBL producing *E. coli* and *K. pneumoniae* isolates. These results highlight the urgent need for AMR strengthened surveillance and antimicrobial stewardship programs in Cambodia.

## Highlights

• High rate of 3 GC and carbapenem resistant Enterobacterales in Cambodia.
• KLUB/CTX-M-1 class predominates among CTX-M-type β-lactamases.
• *Bla*_NDM_ and *bla*_OXA-48_ are the main circulating carbapenemase genes.
• High prevalence of colistin resistance in ESBL producing *E. coli* and *K. pneumoniae.*
• Frequent co-occurrence of multiple beta-lactamase genes.

## 1 Introduction

Antimicrobial resistance (AMR) is a leading cause of mortality worldwide, with the highest burden observed in low-resource settings [1]. The increase of AMR, particularly in Asia, and the lack of new antibiotics limit the prescribing choice for the treatment [2,3]. *Klebsiella pneumoniae* (K. *pneumoniae*) and *Escherichia coli* (*E. coli*) are the main extended-spectrum beta-lactamase-producing Enterobacterales (ESBL-PE), conferring resistance to third-generation cephalosporins (3 GC), have spread worldwide, and are now endemic in several countries [4,5]. Furthermore, for several years, the emergence of Carbapenemase-Producing Enterobacterales (CPE) has become a serious issue for both community-acquired infections (CAI) and healthcare-associated infections (HAI) [6,7]. CPE are, with colistin-resistant bacteria, one of the most problematic multidrug-resistant organisms and are classified as urgent threat by the World Health Organization (WHO) [8].

In Cambodia, emerging AMR remains relatively under-characterized. However, the prevalence of ESBL-producing bacteria has increased significantly over the years, predominantly associated with *E. coli*. The carriage of carbapenemase- and ESBL-producing *E. coli* and *K. pneumoniae* in both humans and livestock are widespread, with ESBL-PE carriage rates reaching up to 92.8 % in children. Additionally, colistin resistance variants, including mcr-1, mcr-3, and mcr-8.2, have been detected [[9], [10], [11], [12], [13], [14], [15]]. CTX-M is the most prevalent ESBL type worldwide, with CTX-M-15 being the dominant variant reported across Southeast Asia, including Cambodia, in healthy individuals, CAI, HAI, and livestock reservoirs [13,16,17]. Most of the studies in Cambodia dealing with CPE concern fecal carriage, but more recently a multicenter study described a worrying rate of 12 % of isolates carrying carbapenemase resistance genes in Gram-negative pathogens isolated from positive blood cultures in six hospitals [18]. With the indiscriminate use of antibiotics in food production, human and veterinary medicine in Cambodia, further studies are needed to understand the spread of AMR and emergence of highly resistant bacteria in a one-health approach in order to identify rapid and appropriate intervention to prevent a post antibiotic era [19]. A comprehensive review providing an overview of published AMR data from Cambodia was published in 2019 [9]. In this context, our study aims to supplement this review, evaluate the current state of AMR, and describe the resistance mechanisms of the main Enterobacterales responsible for various infections in Cambodia.

## 2 Material and methods

### 2.1 Clinical isolate collection from patient samples

**Fig. 1:**
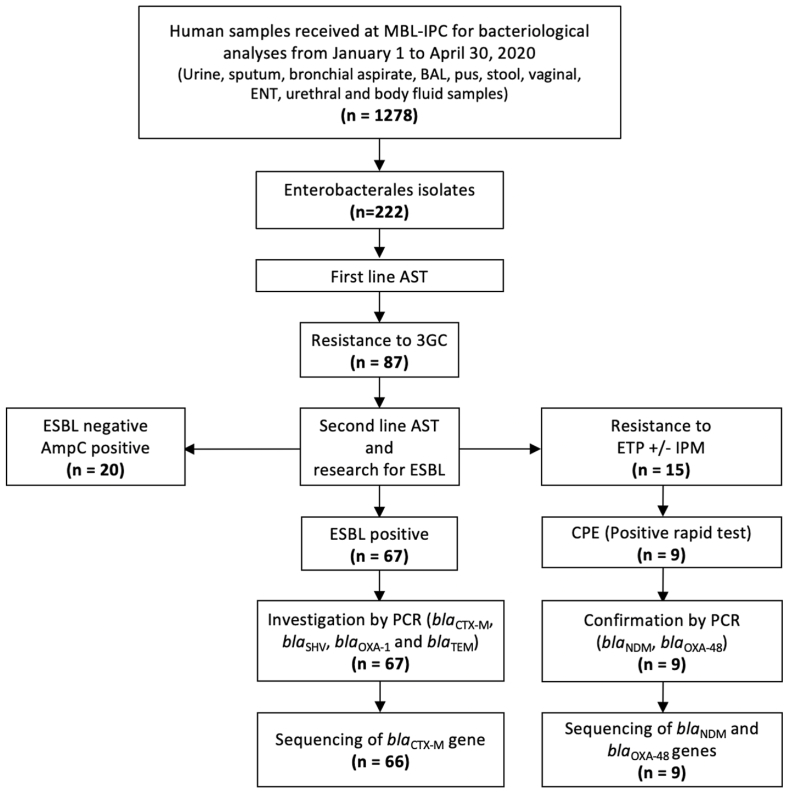
Flow chart of bacterial isolates. 3 GC: Third Generation Cephalosporin; AmpC: Ampicillin Class C; AST: Antimicrobial Susceptibility Testing; BAL: Bronchoalveolar Lavage; CPE: Carbapenemase Producing Enterobacterales; ENT: Ear Nose Throat; ESBL: Extended Spectrum Beta-Lactamase; ETP: Ertapenem; IMP: Imipenem; MBL-IPC: Medical Biology Laboratory - Institut Pasteur du Cambodge; PCR: Polymerase Chain Reaction.

A cross-sectional study was conducted using data from the 1278 bacteriological samples collected from January 1 to April 30, 2020 at the Medical Biology Laboratory of the Institut Pasteur du Cambodge (MBL-IPC) (Fig. 1). Clinical data and information were collected for all patients as part of the laboratory's daily diagnostic mission.

All the samples have been inoculated on specific media and incubated at 35 ± 2 °C between 24 and 48 h according to the specimen type. Growth cultures were undergone bacterial species identification using matrix-assisted laser desorption/ionization time of flight mass spectrometry (Maldi-TOF MS) (Bruker Daltonics, Bremen, Germany) and Antimicrobial Susceptibility Testing (AST).

### 2.2 Antimicrobial susceptibility testing

AST of Enterobacterales isolates were performed following 2019 EUCAST guidelines (version V.1.January 0, 2019) for the following antibiotics (Bio-Rad): amoxicillin (20 μg), amoxicillin-clavulanate (20-10 μg), ticarcillin (75 μg), mecillinam (10 μg), cefotaxime (5 μg), ceftazidime (10 μg), imipenem (10 μg), ertapenem (10 μg), amikacin (30 μg), gentamicin (10 μg), nalidixic acid (30 μg), ciprofloxacin (5 μg), levofloxacin (5 μg), co-trimoxazole 1,25-23,75 μg), nitrofurantoin (100 μg), and fosfomycin (200 μg) [20].

In case of resistance to 3 GC, a second-line antibiotic panel was performed including ticarcillin-clavulanate (75-10 μg), piperacillin-tazobactam (30-6 μg), aztreonam (30 μg), cefepime (30 μg) and tigecycline (15 μg) (Bio-Rad).

The phenotypic detection of ESBL and high-level Ampicillin Class C (AmpC) expression was performed by double-disk synergy test (DDST): disks containing 3 GC (including cefotaxime 5 μg, ceftazidime 10 μg) and cefepime (30 μg) were applied on both Mueller-Hinton plates and Mueller-Hinton agar supplemented with 250 mg/L cloxacillin 30 mm next to amoxicillin-clavulanic acid disk (20-10 μg).

In addition, all the strains resistant to imipenem and/or ertapenem have been tested for carbapenemase production using rapid NG-Test CARBA 5 (NG-BIOTECH) that is able to detect the five main carbapenemase KPC, OXA-48-like, VIM, IMP, and NDM.

The colistin susceptibility testing was performed using the minimum inhibitory concentration (MIC) using broth microdilution according to Clinical Laboratories Standards Institute guidelines [15,21].

### 2.3 Molecular characterization of resistance genes

Characterization of several beta-lactamase genes (*bla*_CTX-M_*, bla*_SHV_*, bla*_OXA-1_ and *bla*_TEM_) was performed by PCR for all strains resistance to 3 GC. From Dallenne et al., primer sets “Multiplex I TEM, SHV, OXA-1-like” was used to amplify *bla*_TEM_, *bla*_SHV_*, bla*_OXA-1_ and primer sets “Multiplex II CTX-M group 1, 2, 9, 8/25” sets were used to amplify *bla*_CTX-M_ [22]. Furthermore, all the strains resistant to at least one carbapenem with a positive rapid test for carbapenemase detection have been confirmed for the presence of different genes encoding for carbapenemase by PCR [23]. PCR products were sequenced for all the CTX-M, NDM, OXA-48-like producing bacteria (Macrogen, Korea), sequencing data were analyzed online using MEGA12 software [24] and iTOL version 6 software was used for tree editing [25].

### 2.4 Statistical analysis

Comparison of categorical variables was performed using Chi-squared or Fisher exact test. A *P* value < 0.05 was considered significant.

## 3 Results

### 3.1 Enterobacterales isolates and epidemiological data

During the study period, a total of 1278 specimens were collected, and 242 AST of Enterobacterales have been performed, representing a total of 222 non-duplicate isolates as some patients have been sampled several times (Fig. 1). The most frequently isolated Enterobacterales*,* among the 11 detected species, were *E. coli* (n = 101; 45.5 %), *K. pneumoniae* (n = 53; 23.9 %), and *Salmonella* Paratyphi A (n = 22; 9.9 %). Of the 222 Enterobacterales isolates, 34.2 % (n = 76) were collected from urine, 29.7 % (n = 66) from respiratory samples (including sputum, bronchial aspirate and bronchoalveolar lavage), 14.0 % (n = 31) from blood culture and 11.7 % (n = 26) from pus. The distribution of the Enterobacterales species according to specimen type is described in Table 1. The most prevalent species in urine, respiratory samples and blood cultures were respectively *E. coli* (86.8 %), *K. pneumoniae* (57.6 %) and *Salmonella* Paratyphi A (71.0 %).

**Table 1:** Number of isolates according to bacterial species and specimen type.

Species	GLASS	*Urine*	*Respiratory*	*Blood*	*Pus*	*Stool*	*Vaginal*	*ENT*	*Urethral*	*Fluid*	TOTAL
*Escherichia coli*	Yes	66	11	3	15	0	3	2	1	0	**101 (45.5 %)**
*Klebsiella pneumoniae*	Yes	5	38	3	3	0	1	2	0	1	**53 (23.9 %)**
*Salmonella* Paratyphi A	Yes	0	0	22	0	0	0	0	0	0	**22 (9.9 %)**
*Enterobacter cloacae* complex	No	1	11	0	2	0	0	0	0	0	**14 (6.3 %)**
NTS sp.	Yes	0	0	0	0	12	0	0	0	0	**12 (5.4 %)**
*Morganella morganii*	No	0	2	0	4	0	1	0	0	0	**7 (3.2 %)**
*Proteus mirabilis*	No	3	2	0	2	0	0	0	0	0	**7 (3.2 %)**
*Salmonella* Typhi	Yes	0	0	2	0	0	0	0	0	0	**2 (0.9 %)**
*Serratia marcescens*	No	0	1	1	0	0	0	0	0	0	**2 (0.9 %)**
*Citrobacter koseri*	No	1	0	0	0	0	0	0	0	0	**1 (0.5 %)**
*Klebsiella oxytoca*	No	0	1	0	0	0	0	0	0	0	**1 (0.5 %)**
**TOTAL**		**76 (34.2 %)**	**66 (29.7 %)**	**31 (14.0 %)**	**26 (11.7 %)**	**12 (5.4 %)**	**5 (2.3 %)**	**4 (1.8 %)**	**1 (0.5 %)**	**1 (0.5 %)**	**222**

ENT: Ear Nose Throat; NTS: Non-typhoidal *Salmonellae*, GLASS: WHO Global antimicrobial resistance and use surveillance system.

Among the 222 patients, 118 were female (53.2 %) and 104 were male (46.8 %). The age range was 1–89, with a median of 56 years (Supplementary data S1).

### 3.2 Antimicrobial susceptibility patterns to main antibiotics

The result of first-line AST is summarized in Table 2. Among the 222 isolates, 72.9 % were resistant to amoxicillin. If we select only *E. coli* isolates, we can observe a high resistance rate to penicillin with 94.1 % of resistance to amoxicillin and 48.5 % of resistance to the association amoxicillin-clavulanate.

**Table 2:** Resistance rates to main antibiotics (n = 222).

Antibiotic family	Penicillins			3 GC		Carbapenems		Aminoglycosides		Quinolones			Others		
Drug	AMX	AMC	TIC	CAZ	CTX	ETP	IPM	AN	GM	NA	CIP	LEV	SXT	FT^1^	FOS^1^
*Escherichia coli*	95/101 94,1 %	49/101 48,5 %	90/101 89,1 %	61/101 60,4 %	61/101 60,4 %	7/101 6,9 %	3/101 3,0 %	0/101 0,0 %	31/101 30,7 %	82/101 81,2 %	72/101 71,3 %	72/101 71,3 %	80/101 79,2 %	0/66 0,0 %	0/66 0,0 %
*Klebsiella pneumoniae*		21/53 49,6 %		20/53 37,7 %	20/53 37,7 %	7/53 13,2 %	3/53 5,7 %	1/53 1,9 %	8/53 15,1 %	24/53 45,3 %	23/53 43,4 %	23/53 43,4 %	19/53 35,8 %	NA	NA
*Salmonella* paratyphi A	0/22 0,0 %	0/22 0,0 %	0/22 0,0 %	0/22 0,0 %	0/22 0,0 %	0/22 0,0 %	0/22 0,0 %	0/22 0,0 %	0/22 0,0 %	22/22 100,0 %			0/22 0,0 %	NA	NA
*Enterobacter cloacae* complex			6/14 42,9 %	3/14 21,4 %	3/14 21,4 %	1/14 7,1 %	1/14 7,1 %	0/14 0,0 %	4/14 28,6 %	6/14 42,9 %	6/14 42,9 %	6/14 42,9 %	8/14 57,1 %	NA	NA
NTS sp.	5/12 41,7 %	1/12 8,3 %	5/12 41,7 %	1/12 8,3 %	1/12 8,3 %	0/12 0,0 %	0/12 0,0 %	0/12 0,0 %	1/12 8,3 %	2/12 16,7 %	2/12 16,7 %	2/12 16,7 %	2/12 16,7 %	NA	NA
*Morganella morganii*			1/7 14,3 %	1/7 14,3 %	1/7 14,3 %	0/7 0,0 %	0/7 0,0 %	0/7 0,0 %	3/7 42,9 %	4/7 57,1 %	4/7 57,1 %	4/7 57,1 %	5/7 71,4 %	NA	NA
*Proteus mirabilis*	5/7 71,4 %	0/7 0,0 %	5/7 71,4 %	0/7 0,0 %	1/7 14,3 %	0/7 0,0 %	0/7 0,0 %	0/7 0,0 %	2/7 28,6 %	4/7 57,1 %	3/7 42,9 %	3/7 42,9 %	4/7 57,1 %	NA	NA
*Salmonella* typhi	0/2 0,0 %	0/2 0,0 %	0/2 0,0 %	0/2 0,0 %	0/2 0,0 %	0/2 0,0 %	0/2 0,0 %	0/2 0,0 %	0/2 0,0 %	2/2 100,0 %			0/2 0,0 %	NA	NA
*Serratia marcescens*			0/2 0,0 %	0/2 0,0 %	0/2 0,0 %	0/2 0,0 %	0/2 0,0 %		0/2 0,0 %	0/2 0,0 %	0/2 0,0 %	0/2 0,0 %	0/2 0,0 %	NA	NA
*Citrobacter koseri*		0/1 0,0 %		0/1 0,0 %	0/1 0,0 %	0/1 0,0 %	0/1 0,0 %	0/1 0,0 %	0/1 0,0 %	0/1 0,0 %	0/1 0,0 %	0/1 0,0 %	0/1 0,0 %	NA	NA
*Klebsiella oxytoca*		0/1 0,0 %		0/1 0,0 %	0/1 0,0 %	0/1 0,0 %	0/1 0,0 %	0/1 0,0 %	0/1 0,0 %	0/1 0,0 %	0/1 0,0 %	0/1 0,0 %	0/1 0,0 %	NA	NA
**TOTAL**	**105/144** **72,9 %**	**71/199** **35,7 %**	**107/167** **64,1 %**	**86/222** **38,7 %**	**87/222** **39,2 %**	**15/222** **6,8 %**	**7/222** **3,2 %**	**1/220** **0,5 %**	**49/222** **22,1 %**	**146/222** **65,8 %**	**110/198** **55,6 %**	**110/198** **55,6 %**	**118/222** **53,2 %**	**0/66** **0,0 %**	**0/66** **0,0 %**

3 GC: third-generation cephalosporin, NTS: Non-typhoidal *Salmonellae*, AMX: amoxicillin; AMC: amoxicillin-clavulanate; TIC: ticarcillin; CAZ: ceftazidime; CTX: cefotaxime; ETP: ertapenem; IPM: imipenem; AN: amikacin; GM: gentamicin; NA: nalidixic acid; CIP: ciprofloxacin; LEV: levofloxacin: SXT: Cotrimoxazole; FT: nitrofurantoin; FOS: fosfomycin.

NA: EUCAST breakpoints for FT and FOS are available only for *E. coli*.

1 FT and FOS have been tested only for isolates from urine samples.

87 isolates (39.2 %) were resistant to at least one 3 GC with a significantly higher proportion for *E. coli* (n = 61; 60.4 %) than *K. pneumoniae* (n = 20; 37.7 %) (*p-value* = 0.007). For other species, the resistance rates to 3 GC are respectively 21.4 % (n = 3), 8.3 % (n = 1) and 14.3 % (n = 1) for *Enterobacter cloacae* complex, Non-typhoidal *Salmonellae,* and both *Morganella morgannii* and *Proteus mirabilis*. *Salmonella* Typhi and Paratyphi A are spared for resistance to beta-lactams with no resistance to amoxicillin but show a rate of resistance to nalidixic acid of 100 %.

15 isolates (6.8 %) were resistant to ertapenem, including 7 (3.2 %) with associated resistance to imipenem. The ratio of resistance to ertapenem is higher for *K. pneumoniae* (n = 7; 13.2 %) than *E. coli* (n = 7: 6.9 %) but without significant difference (*p-value* = 0.241).

49 isolates (22.1 %) of Enterobacterales are resistant to gentamycin, while only one *K. pneumoniae* isolate (0.5 %) is resistant to amikacin.

The level of resistance to quinolones is important with 65.8 % (n = 146) of resistance to nalidixic acid and 55.6 % (n = 110) of resistance to fluoroquinolones. Co-trimoxazole presents a level of resistance estimated at 53.2 % (n = 118). The resistance rate of *E. coli* to nalidixic acid, fluoroquinolones and co-trimoxazole are respectively 81.2 %, 71.3 % and 79.2 % and are significantly higher (*p-value* < 0,001) than for *K. pneumoniae* (respective 45.3 %, 43.4 %, 35.8 %).

Overall, no resistance has been detected in all *E. coli* isolates for nitrofurantoin and fosfomycin that have been tested on strains isolated from urine samples.

### 3.3 Resistance mechanisms and antimicrobial susceptibility patterns to second-line antibiotics

All the 87 isolates resistant to 3 GC have been tested for a second line AST and the resistance mechanisms have been investigated. The results summarized in Table 3. 20 isolates were considered phenotypically positive for isolated AmpC (9.0 % of the overall population) (including 6 isolates resistant to ertapenem but susceptible to imipenem, with a negative rapid test and negative PCR for carbapenemase detection). Respectively 11.9 % (12/101) and 13.2 % (7/53) of *E. coli* and *K. pneumoniae* isolates were positive for AmpC. 67 strains were identified as ESBL-PE (30.2 %), with a significantly higher rate (*p-value* = 0,004) of ESBL among *E. coli* (49/101; 48.5 %) than *K. pneumoniae* (13/53; 24.5 %). The remaining ESBL-PE belongs to *E*. *cloacae* (3/14; 21.4 %), *P*. *mirabilis* (1/7; 14.3 %) and *Salmonella* spp (1/12; 8.4 %). 9 ESBL-PE presented resistant to ertapenem±imipenem as well and have been detected positive for carbapenemase by rapid test (4.1 %). These 9 CPE pertained to *E. coli* (n = 5), *K. pneumoniae* (n = 3) and *E*. *cloacae* complex (n = 1) (Table 4).

**Table 3:** Resistance rates to second-line antibiotics (n = 87).

	TCC	TZP	FEP	ATM	TGC∗
High level AmpC (n = 20)	20/20	18/20	6/20	8/20	0/12
	100,0 %	90,0 %	30,0 %	40,0 %	0,0 %
ESBL (n = 58)	57/58	35/58	58/58	55/58	0/44
	98,3 %	60,3 %	100,0 %	94,8 %	0,0 %
ESBL + CPE (n = 9)	9/9	9/9	9/9	9/9	0/5
	100,0 %	100,0 %	100,0 %	100,0 %	0,0 %
**TOTAL**	**85/87** **97,7 %**	**63/87** **72,4 %**	**74/87** **85,1 %**	**73/87** **83,9 %**	**0/61** **0,0 %**

AmpC: ampicillin class C; ATM: aztreonam; CPE: carbapenemase-producing Enterobacterales; ESBL: extended-spectrum beta-lactamase; FEP: cefepime; TCC: ticarcillin-clavulanate; TGC: tigecyclin.

TZP: piperacillin-tazobactam.

∗ For TGC, only *E. coli* isolates have been tested, as mentioned in 2019 EUCAST guidelines.

**Table 4:** Description of CPE isolates.

Strain	Species	Sample	Province	Clinic	ETP	IPM	OXA-48	NDM
100103018	*Escherichia coli*	Urine	Takeo	Pollakiuria	R	I	OXA-48-like	
100222013	*Escherichia coli*	Urine	Phnom Penh	Dysuria	R	I	OXA-181	
100421004	*Escherichia coli*	Urine	Phnom Penh	Recurrent UTI	R	I	OXA-48-like	
110306093	*Escherichia coli*	BAL	Phnom Penh	Pneumonia	R	S	OXA-48-like	
200413010	*Escherichia coli*	Pus	Phnom Penh	Necrotic wound	R	S	OXA-48-like	
110402004	*Klebsiella pneumoniae*	Sputum	Kandal	md	R	R	OXA-181	NDM-4
110418034	*Klebsiella pneumoniae*	Sputum	Phnom Penh	Pneumonia	R	I	OXA-48-like	
200309026	*Klebsiella pneumoniae*	Pus	Phnom Penh	Pus from belly	R	R	OXA-181	NDM-4
110418019	*Enterobacter cloacae* complex	Sputum	Kandal	md	R	R		NDM-5

BAL: bronchoalveolar lavage; UTI: urinary tract infection; ETP: ertapenem; IPM: imipenem; R: resistant; I: intermediate; S: susceptible; md: missing data.

Resistance to ticarcillin-clavulanate is very high (n = 85; 97.7 %) regardless of the resistance mechanism to 3 GC. Piperacillin-tazobactam is significantly more active on ESBL strains (*p-value* = 0.014), in contrast to cefepime and aztreonam which are more active on AmpC isolates (*p-value* < 0.001) (Table 3). CPE isolates are resistant to all second line antibiotics except tigecycline that remains the only active drug on *E. coli*.

Colistin susceptibility testing was performed on 62 ESBL±CPE isolates (49 *E. coli* and 13 *K. pneumoniae)*. Four strains revealed colistin-resistant phenotypes, representing a rate of 6.5 % among the isolated ESBL producing *E. coli* and *K. pneumoniae*, with respectively 4.1 % (2/49) and 15.4 % (2/13) for *E. coli* and *K. pneumoniae*. They all exhibited MIC equal to 4–8 mg/ml for *E. coli* and to 8 and 32 mg/ml for *K. pneumoniae*.

### 3.4 Molecular characterization of resistance

**Fig. 2:**
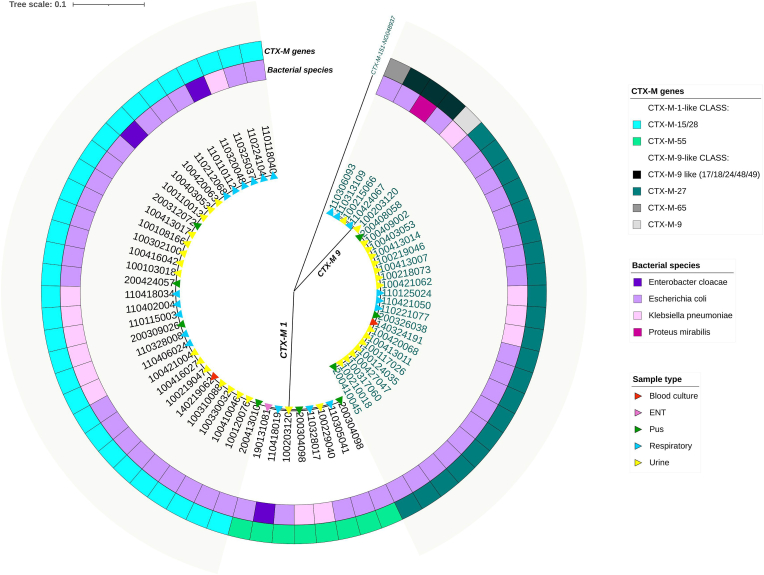
Maximum Likelihood tree based on CTX-M sequences (n = 65). ENT: Ear Nose Throat. CTX-M-151 sequence is used as outgroup (NG048937).

The 67 ESBL-PE ± CPE isolates have been tested for ESBL molecular characterization by screening of *bla*_CTX-M_ genes. PCR products were sequenced to determine the genotypes and deposited in Genbank database (Supplementary data S1). *bla*_CTX-M_ genes pertaining to the KLUB/CTX-M-1 (*bla*_CTX-M-15-like_, *bla*_CTX-M-55_) and KLUY/CTX-M-9 (*bla*_CTX-M-9_, *bla*_CTX-M-9-like_, *bla*_CTX-M-27_, *bla*_CTX-M-65_) classes were the most common and were present in 98.5 % of the isolates (66/67), mainly *bla*_CTX-M-15-like_ (31/66; 47 %) and *bla*_CTX-M-27_ (21/66; 31.8 %) (Fig. 2). Other beta-lactamase genes were frequently associated with ESBL producing isolates, with *bla*_TEM_ as the most frequent (27/67; 40.3 %), followed by *bla*_OXA-1_ (21/67; 31.3 %) and *bla*_SHV_ (12/67; 17.9 %) (Supplementary data S2).

9 bacterial isolates, resistant to ertapenem±imipenem, have been detected positive by NG-Test CARBA 5 (NG-BIOTECH) for OXA-48-like and/or NDM carbapenemase. All positive isolates have been confirmed by PCR for selected carbapenemase genes *bla*_NDM_ and *bla*_OXA–48_ and PCR products were sequenced to identify the respective variants and deposited in Genbank database (Supplementary data S1). Carbapenemase producing *E. coli* and *K. pneumoniae* isolates were all harboring *bla*_OXA–48_ gene, and results of sequencing showed the presence of 2 different variants: *bla*_OXA–181_ and *bla*_OXA–48-like_ (identical to *bla*_OXA–48/505/1306_). Only *K. pneumoniae* isolates (n = 2) were positive for *bla*_NDM-4_, and both were positive for *bla*_OXA–181_ as well. The only carbapenemase producing isolate of *E*. *cloacae* complex was carrying *bla*_NDM-5_ gene (Table 4).

## 4 Discussion

This study describes the phenotypic and genotypic resistance profiles of Enterobacterales isolates involved in human infections (mainly *E. coli* and *K. pneumoniae*) analyzed during a four-month cross-sectionnal study at the Medical Biology Laboratory of the Institut Pasteur du Cambodge, Phnom Penh, Cambodia.

Our findings align with the results reported by *Reed* and al. in 2019, and are closely similar to those from *Yek* and al. in 2024 [9,18]. Regarding resistance to 3 GC, while *K. pneumoniae* exhibited a higher resistance (64.4 %) than *E. coli* (46.4 %) in 2019, our study reports a reversal, with *E. coli* showing a higher resistance rate than *K. pneumoniae* (60.4 % versus 37.7 % respectively). This trend is consistent with the 2024 study, which reported 3 GC resistance rates of 78 % for *E. coli* and 53 % for *K. pneumoniae*. This discrepancy may be attributed to differences in study design, as most studies included in the Reed's review focused on specific sample types or patient populations, whereas our study encompasses all sample types from all patients. Notably, some individual studies from the Reed's review also reported higher 3 GC resistance in *E. coli* than in *K. pneumoniae* [26].

Additionally, while the Reed's review identified only one *K. pneumoniae* isolate resistant to meropenem [9,27], we report a significant increase in carbapenem resistance, with rates of 6.9 % in *E. coli* and 13.2 % in *K. pneumoniae*, comparable to the findings of *Yek* et al., who reported 4.9 % and 14 %, respectively. In contrast, a four-year retrospective study conducted in our laboratory between 2012 and 2015 detected no carbapenem resistance [10], highlighting the rapid emergence of resistance in recent years.

Gentamicin resistance rates in *E. coli* and *K. pneumoniae* are similar between the 2019 and 2024 studies (46.4 % and 44 % for *E. coli*, and 51.1 % and 45 % for *K. pneumoniae*, respectively), while our study reports lower resistance rates of 30.7 % and 15.1 %. This difference may be explained by the fact that previous studies primarily focused on hospitalized patients, who are more likely to have received aminoglycosides as part of their treatment, whereas our study population consisted largely of non-hospitalized patients with CAI, where recent aminoglycoside exposure is less likely.

Regarding fluoroquinolones, all studies report consistently higher resistance rates in *E. coli* (ranging from 55.4 % to 82 %) compared to *K. pneumoniae* (21.9 %–62 %). A similar trend is observed with co-trimoxazole, with *E. coli* resistance rates ranging from 79.2 % to 90 % across the three studies, compared to 35.8 %–50 % in *K. pneumoniae*.

Only amikacin - resistance observed in just one carbapenemase-producing *K. pneumoniae* isolate - along with nitrofurantoin and fosfomycin, remains largely unaffected by antibiotic resistance, as previously described [17].

No resistance to 3 GC has been detected in *Salmonella* Paratyphi A and *Salmonella* Typhi. However, the high level of quinolone resistance is alarming, with all strains showing resistance to nalidixic acid. This finding aligns with multiple studies reporting a significant increase in resistance, with ciprofloxacin resistance rates reaching up to 100 % [18,[28], [29], [30]]. A comprehensive analysis of fluoroquinolone resistance in *Salmonella* Paratyphi A has been published previously [31].

The worrying rise in resistance to 3 GC, carbapenems, and fluoroquinolones between the 2019 review, based on data collected from 2007 to 2018, and our study, alongside that of *Yek* et al., which analyzed data from 2020 to 2021, underscores a rapid and alarming trend. The remarkably increase of carbapenem resistance rates observed over a short period indicate a radical and swift shift in AMR patterns in Cambodia.

ESBL-PE represents 30.2 % of our sample, a result consistent with a previous study conducted at the MBL-IPC between 2012 and 2015, which reported an average ESBL-PE rate of 35.4 % over four years. When analyzing resistance rates by species, our findings align with those from 2012 to 2015, where *E. coli* already exhibited a higher ESBL rate (42.7 %) compared to *K. pneumoniae* (33.7 %) [10]. However, in the present study, only 24.5 % of *K. pneumoniae* isolates were ESBL producers. Rather than reflecting a true decrease in ESBL-producing *K. pneumoniae*, this discrepancy is primarily due to differences in study design.

Considering resistance genes, CTX-M genes encoding for the KLUB/CTX-M-1 and KLUY/CTX-M-9 classes are present in nearly all isolates, with *bla*_CTX-M-15-like_, *bla*_CTX-M-55_, and *bla*_CTX-M-27_ being the predominant types. This finding aligns with previous studies conducted in the Greater Mekong region and Cambodia, except for the absence of *bla*_CTX-M-14_ in our study [14,16,32]. As previously described, CTX-M carriage was significantly associated with resistance to fluoroquinolones, gentamycin, and cotrimoxazole [16,17].

The presence of CPE in Cambodia has been previously documented, primarily in fecal carriage among humans. However, reports of CPE involvement in clinical infections remain scarce [13,14,18,32,33]. The previous study conducted at MBL-IPC between 2012 and 2015 did not report any carbapenem-resistant isolates [10]. Furthermore, detecting three carbapenemase-producing *E. coli* strains from urine samples of walk-in patients with no history of hospitalization underscores the role of CPE in CAI.

PCR products sequencing revealed that four carbapenemase producing *E. coli* isolates carried a *bla*_OXA–48-like_ gene, while one isolate harbored *bla*_OXA–181_ gene. Additionally, among the three carbapenemase producing *K. pneumoniae* isolates, one carried the *bla*_OXA–48-like_ gene, while the two others co-harbored *bla*_OXA–181_ and *bla*_NDM–5_. The co-existence of genes encoding for at least two classes of carbapenemase in *K. pneumoniae* has been reported worldwide [34]. Notably, we did not detect any *K. pneumoniae* isolates producing KPC, despite its widespread global prevalence [34]. Finally, we identified one *E*. *cloacae* isolate harboring *bla*_NDM–5_.

Colistin, or Polymyxin E, is active against Gram-negative bacteria especially the Enterobacterales order [35]. In this study, we identified 4 colistin resistant isolates and our team recently revealed that the two *E. coli* isolates have *mcr-1* variant and one *K. pneumoniae* has *mcr-8.2* whereas the isolates revealed different chromosomic mutations on *mgrB, pmrA, pmrB pmrD or crrB* but not on *phoP and phoQ* [15]. Colistin resistance is observed in 4.1 % of ESBL producing *E. coli* isolates and 15.4 % of ESBL producing *K. pneumoniae* isolates. Although human data for Cambodia are limited, a study from 2013 report colistin resistance rates in bloodstream infections of 0.8 % and 3.1 % for *E. coli* and *K. pneumoniae* respectively [36]. These rates are not comparable to our study as we have investigated colistin susceptibility only in ESBL producing isolates. However, they are comparable to previous findings from our laboratory, where resistance rates were respectively 3.6 % and 25.6 % for *E. coli* and *K. pneumoniae*, primarily isolated from urinary tract infections [15]. These 4 isolates are ESBL-PE and it is known that selective pressure with β-lactams leading to the acquisition of β -lactamase genes may therefore be responsible for co-selection of colistin resistance [35]. Especially, other authors identified CTX-M-15 in several colistin-resistant *K. pneumoniae* [37].

Our study has some limitations. Phylogenetic group determination of isolates was not included. As a future perspective, genotyping both resistant and susceptible Enterobacterales circulating in Cambodia would be valuable, particularly by determining *E. coli* phylogroups and *K. pneumoniae* sequence types using multilocus sequence typing or next-generation sequencing. Another limitation is that most patients from MBL-IPC reside in Phnom Penh, which may not be fully representative of the entire Cambodian population. Additionally, data collection varied across healthcare centers, and a study with a clear distinction between CAI and HAI would provide a more comprehensive understanding of the situation.

## 5 Conclusion

Our study confirms the alarming prevalence of ESBL-producing *E. coli* and *K. pneumoniae*, with CTX-M genes detected in nearly all ESBL isolates, mostly CTX-M-15 like, CTX-M-27 and CTX-M-55. We also highlight a recent and significant increase in carbapenem resistance, with a high prevalence of carbapenemase-producing Enterobacterales (NDM and OXA-48) causing human infections, including community-acquired cases. The spread of carbapenem resistant isolates represents a serious global public health threat and has led to the reuse of colistin as a last-resort treatment. However, we report colistin resistance in ESBL-producing *E. coli* and *K. pneumoniae* in Cambodia.

Recently, Cambodia implemented the GLASS (Global Antimicrobial Resistance Surveillance System) program that bring data from all Cambodian provinces. Expanding data collection across multiple hospitals and laboratories from different provinces, such as the recent study on bloodstream infections [18], would help establish national guidelines for multidrug-resistant bacterial infections and implement necessary interventions to prevent an even greater increase in antibiotic resistance level.

## CRediT authorship contribution statement

**Gauthier Delvallez:** Writing – review & editing, Writing – original draft, Validation, Supervision, Resources, Investigation, Formal analysis, Data curation, Conceptualization. **Sokleaph Cheng:** Writing – review & editing, Resources, Investigation, Conceptualization. **Florian Girond:** Writing – review & editing. **Sidonn Krang:** Writing – review & editing. **Soda Meng:** Writing – review & editing, Investigation. **Puthea Nop:** Writing – review & editing, Investigation. **Seiha Heng:** Writing – review & editing, Investigation. **Samrach Han:** Writing – review & editing, Investigation. **Sokunthy Keo:** Writing – review & editing, Investigation. **Kunthea Kong:** Writing – review & editing, Investigation. **Anne-Laure Bañuls:** Writing – review & editing, Resources, Conceptualization. **Mallorie Hide:** Writing – review & editing, Writing – original draft, Visualization, Validation, Supervision, Resources, Investigation, Formal analysis, Data curation, Conceptualization.

## Ethical considerations

Permission for the study was obtained from the National Ethics Committee for Health Research (NECHR) in Cambodia (Approval letter number 074NECHR).

## Funding source

This work was funded by the project of the International Joint Laboratory on Drug Resistance in Southeast Asia (LMI DRISA, IRD) and IPC (Medical Biology Laboratory of the Institut Pasteur du Cambodge).

## Declaration of competing interest

The authors declare that they have no known competing financial interests or personal relationships that could have appeared to influence the work reported in this paper.
