# Supplementary material for: Prevalence and characterization of third-generation cephalosporin, carbapenem and colistin-resistant Enterobacterales isolated from clinical samples in Cambodia
Source: New Microbes New Infect. 2025 Oct 10;68:101649. doi: 10.1016/j.nmni.2025.101649 (PMC12550295; doi:10.1016/j.nmni.2025.101649)
Supplement: Multimedia component 1 [file mmc1.pdf]

Epidemiological information					ESBL				Carbapenemase						
**Strain ID**	**Sex**	**Age**	**Bacterial Species**	**Sample type**	**ESBL**	**CTX-M**	***blaCTX-M* allele**	***blaCTX-M* Accession Number (Genbank)**	**CPE**	**OXA-48**	***blaOXA-48* allele**	***blaOXA-48* Accession Number (Genbank)**	**NDM**	***bla-NDM* allele**	***bla-NDM* Accession Number (Genbank)**
110402004	M	25	*Klebsiella pneumoniae*	Respiratory	1	1	CTX-M-15-28	PV296261	1	1	OXA-181	PV353855	1	NDM-4	PV353859
200309026	M	69	*Klebsiella pneumoniae*	Pus	1	1	CTX-M-15-28	PV296265	1	1	OXA-181	PV353857	1	NDM-4	PV353858
100103018	F	68	*Escherichia coli*	Urine	1	1	CTX-M-15-28	PV296238	1	1	OXA-48-like	PV353853	0	na	na
100421004	F	75	*Escherichia coli*	Urine	1	1	CTX-M-15-28	PV296252	1	1	OXA-48-like	PV353852	0	na	na
110418034	M	76	*Klebsiella pneumoniae*	Respiratory	1	1	CTX-M-15-28	PV296263	1	1	OXA-48-like	PV353850	0	na	na
200413010	M	49	*Escherichia coli*	Pus	1	1	CTX-M-15-28	PV296267	1	1	OXA-48-like	PV353851	0	na	na
110418019	M	86	*Enterobacter cloacae*	Respiratory	1	1	CTX-M-55-like	PV296300	1	0	na	na	1	NDM-5	PV353860
110306093	F	71	*Escherichia coli*	Respiratory	1	1	CTX-M-65	PV296301	1	1	OXA-48-like	PV353854	0	na	na
100222013	M	67	*Escherichia coli*	Urine	1	0	na	na	1	1	OXA-181	PV353856	0	na	na
100108166	F	48	*Escherichia coli*	Urine	1	1	CTX-M-15-28	PV296239	0						
100110013	M	46	*Escherichia coli*	Urine	1	1	CTX-M-15-28	PV296240	0						
100120076	F	74	*Escherichia coli*	Urine	1	1	CTX-M-15-28	PV296241	0						
100219047	F	21	*Escherichia coli*	Urine	1	1	CTX-M-15-28	PV296242	0						
100302100	F	41	*Escherichia coli*	Urine	1	1	CTX-M-15-28	PV296243	0						
100310088	F	56	*Escherichia coli*	Urine	1	1	CTX-M-15-28	PV296244	0						
100330032	F	58	*Escherichia coli*	Urine	1	1	CTX-M-15-28	PV296245	0						
100403053	M	36	*Enterobacter cloacae*	Urine	1	1	CTX-M-15-28	PV296246	0						
100410046	F	1	*Escherichia coli*	Urine	1	1	CTX-M-15-28	PV296247	0						
100413017	F	38	*Escherichia coli*	Urine	1	1	CTX-M-15-28	PV296248	0						
100416027	F	24	*Escherichia coli*	Urine	1	1	CTX-M-15-28	PV296249	0						
100416042	M	69	*Escherichia coli*	Urine	1	1	CTX-M-15-28	PV296250	0						
100420063	M	80	*Escherichia coli*	Urine	1	1	CTX-M-15-28	PV296251	0						
110110112	M	58	*Escherichia coli*	Respiratory	1	1	CTX-M-15-28	PV296253	0						
110115003	M	88	*Klebsiella pneumoniae*	Respiratory	1	1	CTX-M-15-28	PV296254	0						
110118040	M	74	*Escherichia coli*	Respiratory	1	1	CTX-M-15-28	PV296255	0						
110212068	M	62	*Escherichia coli*	Respiratory	1	1	CTX-M-15-28	PV296256	0						
110224104	M	76	*Escherichia coli*	Respiratory	1	1	CTX-M-15-28	PV296257	0						
110320048	M	65	*Enterobacter cloacae*	Respiratory	1	1	CTX-M-15-28	PV296258	0						
110325037	F	69	*Klebsiella pneumoniae*	Respiratory	1	1	CTX-M-15-28	PV296259	0						

110328008	M	60	*Klebsiella pneumoniae*	Respiratory	1	1	CTX-M-15-28	PV296260	0							
110406024	M	69	*Klebsiella pneumoniae*	Respiratory	1	1	CTX-M-15-28	PV296262	0							
140219062	M	74	*Escherichia coli*	Blood culture	1	1	CTX-M-15-28	PV296264	0							
200312072	F	89	*Escherichia coli*	Pus	1	1	CTX-M-15-28	PV296266	0							
200424057	M	53	*Escherichia coli*	Pus	1	1	CTX-M-15-28	PV296268	0							
100117026	F	61	*Escherichia coli*	Urine	1	1	CTX-M-27	PV296272	0							
100124035	M	70	*Escherichia coli*	Urine	1	1	CTX-M-27	PV296273	0							
100210018	F	60	*Escherichia coli*	Urine	1	1	CTX-M-27	PV296274	0							
100218073	F	80	*Escherichia coli*	Urine	1	1	CTX-M-27	PV296275	0							
100219046	F	37	*Escherichia coli*	Urine	1	1	CTX-M-27	PV296276	0							
100317060	F	45	*Escherichia coli*	Urine	1	1	CTX-M-27	PV296277	0							
100403053	M	36	*Escherichia coli*	Urine	1	1	CTX-M-27	PV296278	0							
100409002	F	6	*Escherichia coli*	Urine	1	1	CTX-M-27	PV296279	0							
100413007	F	69	*Escherichia coli*	Urine	1	1	CTX-M-27	PV296280	0							
100413011	F	31	*Escherichia coli*	Urine	1	1	CTX-M-27	PV296281	0							
100413014	F	28	*Escherichia coli*	Urine	1	1	CTX-M-27	PV296282	0							
100420068	F	78	*Escherichia coli*	Urine	1	1	CTX-M-27	PV296283	0							
100421062	M	1	*Escherichia coli*	Urine	1	1	CTX-M-27	PV296284	0							
100427047	F	29	*Escherichia coli*	Urine	1	1	CTX-M-27	PV296285	0							
110125024	M	46	*Klebsiella pneumoniae*	Respiratory	1	1	CTX-M-27	PV296286	0							
110221077	F	48	*Klebsiella pneumoniae*	Respiratory	1	1	CTX-M-27	PV296287	0							
110421050	M	54	*Klebsiella pneumoniae*	Respiratory	1	1	CTX-M-27	PV296288	0							
140324191	F	36	*Escherichia coli*	Blood culture	1	1	CTX-M-27	PV296289	0							
200326038	F	67	*Escherichia coli*	Pus	1	1	CTX-M-27	PV296290	0							
200408058	F	71	*Escherichia coli*	Pus	1	1	CTX-M-27	PV296291	0							
200410045	M	33	*Escherichia coli*	Pus	1	1	CTX-M-27	PV296292	0							
100203120	F	79	*Escherichia coli*	Urine	1	1	CTX-M-55	PV296293	0							
100229040	F	32	*Escherichia coli*	Urine	1	1	CTX-M-55	PV296294	0							
110305041	M	77	*Escherichia coli*	Respiratory	1	1	CTX-M-55	PV296295	0							
110328017	M	73	*Klebsiella pneumoniae*	Respiratory	1	1	CTX-M-55	PV296296	0							
190131081	M	78	*Escherichia coli*	ENT	1	1	CTX-M-55	PV296297	0							
200304098	F	84	*Escherichia coli*	Pus	1	1	CTX-M-55	PV296298	0							
200304098	F	84	*Klebsiella pneumoniae*	Pus	1	1	CTX-M-55	PV296299	0							

100203120	F	79	*Klebsiella pneumoniae*	Urine	1	1	CTX-M-9	PV296302	0						
100215066	F	73	*Proteus mirabilis*	Urine	1	1	CTX-M-9-like	PV296269	0						
110313109	M	55	*Escherichia coli*	Respiratory	1	1	CTX-M-9-like	PV296270	0						
110424067	M	72	*Escherichia coli*	Respiratory	1	1	CTX-M-9-like	PV296271	0						
220407045	M	20	*Salmonella sp*	Stool	1	1	ND	ND	0						
100102011	F	67	*Escherichia coli*	Urine	0				0						
100102092	F	61	*Escherichia coli*	Urine	0				0						
100103087	F	40	*Escherichia coli*	Urine	0				0						
100106189	F	62	*Escherichia coli*	Urine	0				0						
100108167	F	44	*Escherichia coli*	Urine	0				0						
100108171	M	34	*Escherichia coli*	Urine	0				0						
100110121	F	38	*Escherichia coli*	Urine	0				0						
100110122	F	75	*Escherichia coli*	Urine	0				0						
100110122	F	75	*Klebsiella pneumoniae*	Urine	0				0						
100117066	F	8	*Escherichia coli*	Urine	0				0						
100118004	F	33	*Escherichia coli*	Urine	0				0						
100203042	F	34	*Klebsiella pneumoniae*	Urine	0				0						
100206082	F	34	*Escherichia coli*	Urine	0				0						
100211122	F	47	*Escherichia coli*	Urine	0				0						
100217119	F	73	*Escherichia coli*	Urine	0				0						
100218119	F	53	*Escherichia coli*	Urine	0				0						
100219057	F	58	*Escherichia coli*	Urine	0				0						
100221100	F	65	*Escherichia coli*	Urine	0				0						
100227039	F	68	*Escherichia coli*	Urine	0				0						
100228128	F	34	*Escherichia coli*	Urine	0				0						
100303038	F	77	*Escherichia coli*	Urine	0				0						
100306094	F	46	*Escherichia coli*	Urine	0				0						
100310059	F	25	*Escherichia coli*	Urine	0				0						
100311039	F	46	*Klebsiella pneumoniae*	Urine	0				0						
100323060	F	30	*Escherichia coli*	Urine	0				0						
100330006	F	34	*Escherichia coli*	Urine	0				0						
100330034	M	83	*Escherichia coli*	Urine	0				0						
100330043	F	59	*Proteus mirabilis*	Urine	0				0						

100407038	F	22	*Escherichia coli*	Urine	0				0						
100409049	F	5	*Escherichia coli*	Urine	0				0						
100414031	F	24	*Escherichia coli*	Urine	0				0						
100416040	F	67	*Escherichia coli*	Urine	0				0						
100417031	F	89	*Escherichia coli*	Urine	0				0						
100417032	F	57	*Escherichia coli*	Urine	0				0						
100418029	M	59	*Proteus mirabilis*	Urine	0				0						
100420005	F	66	*Escherichia coli*	Urine	0				0						
100421058	M	38	*Citrobacter koseri*	Urine	0				0						
100421064	M	85	*Klebsiella pneumoniae*	Urine	0				0						
100425019	F	50	*Escherichia coli*	Urine	0				0						
100425034	F	51	*Escherichia coli*	Urine	0				0						
100425037	F	48	*Escherichia coli*	Urine	0				0						
100428042	F	38	*Escherichia coli*	Urine	0				0						
110103096	F	78	*Enterobacter cloacae*	Respiratory	0				0						
110107031	F	64	*Klebsiella pneumoniae*	Respiratory	0				0						
110107043	F	71	*Klebsiella pneumoniae*	Respiratory	0				0						
110108059	F	80	*Enterobacter cloacae*	Respiratory	0				0						
110109035	M	32	*Klebsiella oxytoca*	Respiratory	0				0						
110110108	M	77	*Escherichia coli*	Respiratory	0				0						
110115013	F	76	*Proteus mirabilis*	Respiratory	0				0						
110120031	F	78	*Escherichia coli*	Respiratory	0				0						
110120052	M	81	*Klebsiella pneumoniae*	Respiratory	0				0						
110124045	F	81	*Morganella morganii*	Respiratory	0				0						
110124054	F	48	*Klebsiella pneumoniae*	Respiratory	0				0						
110127143	M	67	*Klebsiella pneumoniae*	Respiratory	0				0						
110205100	M	79	*Enterobacter cloacae*	Respiratory	0				0						
110205100	M	79	*Morganella morganii*	Respiratory	0				0						
110205114	F	87	*Klebsiella pneumoniae*	Respiratory	0				0						
110206028	M	72	*Klebsiella pneumoniae*	Respiratory	0				0						
110206090	M	43	*Klebsiella pneumoniae*	Respiratory	0				0						
110207067	M	36	*Klebsiella pneumoniae*	Respiratory	0				0						
110211117	F	80	*Escherichia coli*	Respiratory	0				0						

110212068	M	62	*Klebsiella pneumoniae*	Respiratory	0				0						
110213042	M	33	*Klebsiella pneumoniae*	Respiratory	0				0						
110213082	M	84	*Klebsiella pneumoniae*	Respiratory	0				0						
110215048	M	58	*Enterobacter aerogenes*	Respiratory	0				0						
110217109	M	41	*Serratia marcescens*	Respiratory	0				0						
110219065	F	20	*Enterobacter cloacae*	Respiratory	0				0						
110220078	M	78	*Klebsiella pneumoniae*	Respiratory	0				0						
110224066	M	71	*Klebsiella pneumoniae*	Respiratory	0				0						
110303016	M	72	*Klebsiella pneumoniae*	Respiratory	0				0						
110307020	M	73	*Klebsiella pneumoniae*	Respiratory	0				0						
110312011	F	78	*Enterobacter cloacae*	Respiratory	0				0						
110316065	M	61	*Enterobacter cloacae*	Respiratory	0				0						
110318011	M	58	*Klebsiella pneumoniae*	Respiratory	0				0						
110318041	M	58	*Klebsiella pneumoniae*	Respiratory	0				0						
110320048	M	65	*Klebsiella pneumoniae*	Respiratory	0				0						
110328010	F	70	*Klebsiella pneumoniae*	Respiratory	0				0						
110330053	M	30	*Klebsiella pneumoniae*	Respiratory	0				0						
110401033	M	63	*Klebsiella pneumoniae*	Respiratory	0				0						
110402005	F	79	*Klebsiella pneumoniae*	Respiratory	0				0						
110406034	M	58	*Klebsiella pneumoniae*	Respiratory	0				0						
110410030	M	77	*Proteus mirabilis*	Respiratory	0				0						
110414022	M	41	*Klebsiella pneumoniae*	Respiratory	0				0						
110414041	M	86	*Klebsiella pneumoniae*	Respiratory	0				0						
110420055	M	85	*Klebsiella pneumoniae*	Respiratory	0				0						
110421069	M	81	*Enterobacter cloacae*	Respiratory	0				0						
110427064	M	47	*Klebsiella pneumoniae*	Respiratory	0				0						
110430056	F	85	*Enterobacter cloacae*	Respiratory	0				0						
140131069	F	23	*Salmonella ser. Paratyphi A*	Blood culture	0				0						
140131071	F	41	*Salmonella ser. Paratyphi A*	Blood culture	0				0						
140210120	M	24	*Salmonella ser. Paratyphi A*	Blood culture	0				0						
140305055	M	23	*Salmonella ser. Paratyphi A*	Blood culture	0				0						
140305086	F	63	*Serratia marcescens*	Blood culture	0				0						
140311055	M	25	*Salmonella ser. Paratyphi A*	Blood culture	0				0						

140319051	F	33	*Salmonella ser. Paratyphi A*	Blood culture	0				0						
140320034	F	46	*Salmonella ser. Paratyphi A*	Blood culture	0				0						
140324127	M	27	*Salmonella ser. Paratyphi A*	Blood culture	0				0						
140324216	F	70	*Klebsiella pneumoniae*	Blood culture	0				0						
140326040	F	28	*Salmonella ser. Paratyphi A*	Blood culture	0				0						
140327022	M	33	*Salmonella ser. Paratyphi A*	Blood culture	0				0						
140327025	F	14	*Salmonella ser. Paratyphi A*	Blood culture	0				0						
140330004	M	30	*Salmonella ser. Paratyphi A*	Blood culture	0				0						
140331029	M	43	*Salmonella ser. Paratyphi A*	Blood culture	0				0						
140402068	F	23	*Salmonella ser. Paratyphi A*	Blood culture	0				0						
140403056	F	55	*Salmonella ser. Paratyphi A*	Blood culture	0				0						
140404031	M	36	*Salmonella ser. Paratyphi A*	Blood culture	0				0						
140404033	M	45	*Salmonella ser. Paratyphi A*	Blood culture	0				0						
140406060	M	60	*Salmonella ser. Typhi*	Blood culture	0				0						
140406061	M	51	*Salmonella ser. Paratyphi A*	Blood culture	0				0						
140407040	M	25	*Salmonella ser. Paratyphi A*	Blood culture	0				0						
140411028	F	36	*Salmonella ser. Paratyphi A*	Blood culture	0				0						
140411034	M	58	*Klebsiella pneumoniae*	Blood culture	0				0						
140415020	F	27	*Salmonella ser. Typhi*	Blood culture	0				0						
140420015	M	33	*Salmonella ser. Paratyphi A*	Blood culture	0				0						
140420069	M	55	*Klebsiella pneumoniae*	Blood culture	0				0						
140421055	M	73	*Escherichia coli*	Blood culture	0				0						
140428046	F	22	*Salmonella ser. Paratyphi A*	Blood culture	0				0						
170430067	M	36	*Klebsiella pneumoniae*	Fluid	0				0						
190206126	F	24	*Klebsiella pneumoniae*	ENT	0				0						
190406021	M	49	*Escherichia coli*	ENT	0				0						
190406021	M	49	*Klebsiella pneumoniae*	ENT	0				0						
200205146	M	57	*Enterobacter aerogenes*	Pus	0				0						
200214077	M	74	*Morganella morganii*	Pus	0				0						
200214077	M	74	*Proteus mirabilis*	Pus	0				0						
200227089	M	51	*Morganella morganii*	Pus	0				0						
200302133	F	34	*Escherichia coli*	Pus	0				0						
200319042	M	36	*Morganella morganii*	Pus	0				0						

200319042	M	36	*Proteus mirabilis*	Pus	0				0						
200324204	M	55	*Morganella morganii*	Pus	0				0						
200402065	F	27	*Escherichia coli*	Pus	0				0						
200403025	M	87	*Escherichia coli*	Pus	0				0						
200404026	M	66	*Escherichia coli*	Pus	0				0						
200407037	F	6	*Escherichia coli*	Pus	0				0						
200414018	F	71	*Escherichia coli*	Pus	0				0						
200416028	F	72	*Enterobacter aerogenes*	Pus	0				0						
200420016	F	64	*Escherichia coli*	Pus	0				0						
200420048	M	73	*Escherichia coli*	Pus	0				0						
200421054	M	55	*Klebsiella pneumoniae*	Pus	0				0						
220109096	M	56	*Salmonella sp*	Stool	0				0						
220113015	M	30	*Salmonella sp*	Stool	0				0						
220121019	F	36	*Salmonella ser. London*	Stool	0				0						
220128064	M	33	*Salmonella sp*	Stool	0				0						
220210135	F	3	*Salmonella sp*	Stool	0				0						
220214125	F	27	*Salmonella sp*	Stool	0				0						
220304066	F	26	*Salmonella sp*	Stool	0				0						
220319022	F	45	*Salmonella sp*	Stool	0				0						
220319048	F	24	*Salmonella sp*	Stool	0				0						
220326048	M	64	*Salmonella sp*	Stool	0				0						
220421014	M	56	*Salmonella sp*	Stool	0				0						
230115011	M	29	*Escherichia coli*	Urethral	0				0						
240108067	F	30	*Escherichia coli*	Vaginal	0				0						
240116038	F	36	*Klebsiella pneumoniae*	Vaginal	0				0						
240116038	F	36	*Morganella morganii*	Vaginal	0				0						
240227024	F	32	*Escherichia coli*	Vaginal	0				0						
240314034	F	28	*Escherichia coli*	Vaginal	0				0						
