# Supplementary material for: Prevalence and characterization of third-generation cephalosporin, carbapenem and colistin-resistant Enterobacterales isolated from clinical samples in Cambodia
Source: New Microbes New Infect. 2025 Oct 10;68:101649. doi: 10.1016/j.nmni.2025.101649 (PMC12550295; doi:10.1016/j.nmni.2025.101649)
Supplement: Multimedia component 2 [file mmc2.docx]

**Supplementary data S2: Co-occurrence of beta-lactamase genes in CTX-M producing Enterobacterales**

| CTX-M class | KLUB/CTX-M-1 | | | | | | | | | | KLUY/CTX-M-9 | | | | | | **TOTAL** |
| --- | --- | --- | --- | --- | --- | --- | --- | --- | --- | --- | --- | --- | --- | --- | --- | --- | --- |
| bla_CTX-M_ gene | CTX-M-15-28-like | | | | | | CTX-M-55 / CTX-M-55-like | | | | CTX-M-9 like / CTX-M-27 / CTX-M-65 | | | | | |  |
| Other beta-lactamase genes | None | *bla*_OXA-1_ | *bla*_TEM_ | *bla*_TEM_ *bla*_OXA-1_ | *bla*_TEM_   *bla*_SHV_ | *bla*_TEM_   *bla*_SHV_ *bla*_OXA-1_ | None | *bla*_TEM_ | *bla*_SHV_ *bla*_OXA-1_ | *bla*_TEM_   *bla*_SHV_ | None | *bla*_TEM_ | *bla*_SHV_ | *bla*_OXA-1_ | *bla*_TEM_   *bla*_SHV_ | *bla*_TEM_   *bla*_SHV_ *bla*_OXA-1_ |  |
| *Escherichia coli* | 8 | 6 | 3 | 5 | 0 | 0 | 2 | 2 | 1 | 0 | 14 | 5 | 0 | 1 | 0 | 1 | 48 |
| *Klebsiella pneumoniae* | 2 | 0 | 0 | 0 | 2 | 3 | 0 | 0 | 1 | 1 | 1 | 0 | 2 | 0 | 1 | 0 | 13 |
| *Enterobacter cloacae* complex | 0 | 1 | 0 | 1 | 0 | 0 | 0 | 1 | 0 | 0 | 0 | 0 | 0 | 0 | 0 | 0 | 3 |
| *Proteus mirabilis* | 0 | 0 | 0 | 0 | 0 | 0 | 0 | 0 | 0 | 0 | 0 | 1 | 0 | 0 | 0 | 0 | 1 |
| **TOTAL** | 10 | 7 | 3 | 6 | 2 | 3 | 2 | 3 | 2 | 1 | 15 | 6 | 2 | 1 | 1 | 1 | 65* |
|  | 31 | | | | | | 8 | | | | 26 | | | | | |  |

* Among the 67 ESBL-producing isolates, 66 harbor a CTX-M gene. Due to a sequencing failure in one *Salmonella* isolate, results are summarized for 65 isolates
